# An Adaptive Weighting Algorithm for Interpolating the Soil Potassium Content

**DOI:** 10.1038/srep23889

**Published:** 2016-04-07

**Authors:** Wei Liu, Peijun Du, Zhuowen Zhao, Lianpeng Zhang

**Affiliations:** 1 Jiangsu Provincial Key Laboratory of Geographic Information Science and Technology, Nanjing University, Nanjing, People’s Republic of China; 2 School of Geodesy and Geometrics, Jiangsu Normal University, Xuzhou, People’s Republic of China

## Abstract

The concept of spatial interpolation is important in the soil sciences. However, the use of a single global interpolation model is often limited by certain conditions (e.g., terrain complexity), which leads to distorted interpolation results. Here we present a method of adaptive weighting combined environmental variables for soil properties interpolation (AW-SP) to improve accuracy. Using various environmental variables, AW-SP was used to interpolate soil potassium content in Qinghai Lake Basin. To evaluate AW-SP performance, we compared it with that of inverse distance weighting (IDW), ordinary kriging, and OK combined with different environmental variables. The experimental results showed that the methods combined with environmental variables did not always improve prediction accuracy even if there was a strong correlation between the soil properties and environmental variables. However, compared with IDW, OK, and OK combined with different environmental variables, AW-SP is more stable and has lower mean absolute and root mean square errors. Furthermore, the AW-SP maps provided improved details of soil potassium content and provided clearer boundaries to its spatial distribution. In conclusion, AW-SP can not only reduce prediction errors, it also accounts for the distribution and contributions of environmental variables, making the spatial interpolation of soil potassium content more reasonable.

The continuous spatial distribution of the soil plays a significant role in the fields of agriculture and environmental management^1^,^2^. Scientists and agricultural managers often require continuous data of soil properties over a region of interest for making informed decisions and justified interpretations. However, such data are usually not readily available and often difficult and expensive to acquire, especially for high-altitude mountainous regions. Moreover, soil property data are usually collected by point sampling. Thus, attribute values at unsampled points require estimation for the generation of spatially continuous data of soil properties. Therefore, spatial interpolation techniques are essential for predicting the continuous data of soil properties for unsampled locations using data from limited point observations.

Existing spatial interpolation methods can be largely classified into three groups^3^: 1) deterministic or non-geostatistical methods (e.g., inverse distance weighting [IDW]); 2) stochastic or geostatistical methods (e.g., universal kriging [UK]); and 3) combined methods (e.g., ordinary kriging [OK] with environmental variables [OK-Geo]). Kriging is a geostatistical interpolation method that provides the best linear unbiased estimation for the interpolation result, but demands the higher data stability, such as meeting a second-order stationary assumption. Non-geostatistical interpolation methods such as IDW interpolation, which assume that each sampling point on the local impact with the increase in distance gradually disappears, do not have the statistical advantage despite its simple operation. However, such methods are often data- or even variable-specific, and their performance depends on many factors^4^. No consistent findings have shown how these factors affect spatial prediction method performance. Some researchers found that kriging method outperformed IDW and other methods because of a careful choice of the optimal number of neighboring points as well as the variogram model and its parameters^3^,^5^,^6^,^7^,^8^,^9^,^10^,^11^; however, the others showed that kriging was not better than the other methods^4^,^12^,^13^,^14^,^15^. Therefore, it is often a challenge to select an appropriate spatial interpolation method for a given dataset.

Machine learning methods have been applied to the fields of data mining and spatial interpolation and have demonstrated their predictive accuracy, e.g., artificial neural networks (ANN), support vector machine (SVM), ensemble learning(EL), and random forest (RF)^10^,^16^,^17^,^18^. Furthermore, ANN and SVM have been applied to daily minimum air temperature and rainfall data in the studies by Gilardi^19^ and Rigol *et al*.^20^. EL and RF were previously applied to the spatial interpolation of environmental variables by Liu^21^ and Li^22^. However, this is a kind of global interpolation model, a simple iteration of which cannot explain the spatial instability of soil properties.

AW-SP, a machine learning paradigm in which multiple learners are trained to solve the same problem, originated from Hansen and Salamon’s work^23^, which showed that the generalization ability of a model system can be significantly improved through use of a number of models, i.e. training many models and then combining their predictions. Since this technology performs remarkably well, it has become a very hot topic in machine learning communities^24^. In contrast to ordinary interpolation approaches that try to use one interpolation model based on training data, AW-SP try to construct a set of interpolation models and combine them. As in the axiom ‘many hands make light work’, the predictive ability of the ensemble is usually significantly better than that of a single model^24^.

The spatial distribution of soil is also greatly affected by environmental features including land use type, soil type, geological type, and slope, etc.^22^,^25^. Soil property data may vary significantly within a short horizontal distance because of the different soil environment, it is difficult to accurately interpolate soil property distributions in the absence of obvious environment features. Therefore, spatial interpolators combining environment features in pedometrics and digital soil have been studied by increasingly more researchers^5^,^25^,^26^,^27^,^28^,^29^,^30^,^31^,^32^. We used the secondary variables in an effort to improve interpolation accuracy.

In this study, we aim to address the following questions: 1) can ensemble of these existing spatial interpolation methods with environmental variables improve the predictive accuracy? 2) can describe spatial distribution pattern of soil potassium content more accurately based on difference environmental variables? To address the two questions, we applied the ensemble learning methods and a number of existing spatial interpolation methods including IDW, OK and the combined methods (e.g., OK combined with environmental variables) to soil potassium content we collected from around the Qinghai Lake in September, 2013.We examined the effects of the performance of AW-SP, IDW, OK and its combined methods (Table 1). Finally, the prediction patterns of the interpolation methods were analyzed based on their prediction maps.

## Results

### Comparison of interpolation performance

To assess the accuracy of AW-SP for interpolating soil potassium content, we compared the performances of AW-SP, IDW, OK, OK-LU, OK-Soil, OK-Grassland, OK-Geology, and OK-Geo. We calculated the mean absolute error (MAE), root mean square error (RMSE), and mean error (ME) as measures of interpolation quality by comparing the predicted and measured values (Table 2). We found that interpolation combined with environmental variables, including AW-SP, OK-LU, OK-Soil, OK-Grassland, and OK-Geo (i.e., excluding OK-Geology) obviously improved interpolation precision. AW-SP in particular was the most accurate method. OK and IDW had similar performance. AW-SP could achieve a slightly better MAE (0.1003) than those of OK-Geo (0.1284), OK-LU (0.1376), OK-Soil (0.1381), OK-Grassland (0.1387), OK (0.1485), IDW (0.1487), and OK-Geology (0.1521). Similarly, the RMSE for AW-SP (0.1374) was the smallest, followed by OK-Geo (0.1732), OK-LU (0.1741), OK-Soil (0.1754), OK-Grassland (0.1797), OK (0.1838), IDW (0.1872), and OK-Geology (0.2022). The ME of AW-SP (0.0000) was much smaller than those of OK-Geo (0.0011), OK-LU (0.0017), OK-Soil (0.0015), OK-Grassland (0.0012), OK (0.0026), OK-Geology (0.0026), and IDW (−0.0030).

### Effects of the exclusion of slope and geology

The prediction errors of the two combined with all environmental variables methods (i.e., AW-SP and OK-Geo) are reduced after the exclusion of slope and geology information in terms of RMAE, RRMSE and ME, although *p-values* change with the methods and with predictive error measurements (Table 3). Overall, the methods without slope and geology information are relatively more accurate than those with slope and geology information.

### Comparison of the interpolated maps

**Figure 1:**
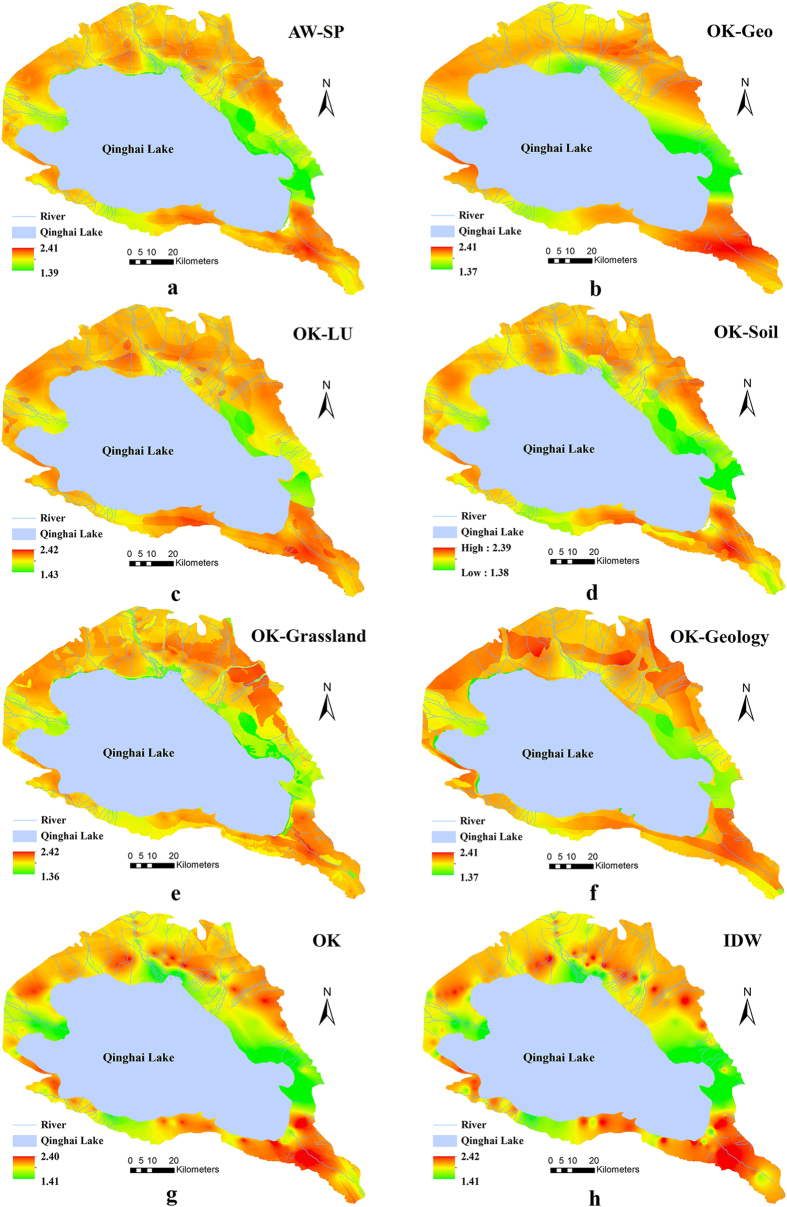
Comparisons of the soil potassium content maps interpolated by (**a**) AW-SP, (**b**) OK-Geo, (**c**) OK-LU, (**d**) OK-Soil, (**e**) OK-Grassland, (**f**) OK-Geology, (**g**) OK and (**h**) IDW. All the maps were generated in ArcGIS10.1, URL: http://www.esrichina-bj.cn/softwareproduct/ArcGIS/.

Applied 8 methods to interpolate the soil potassium content (i.e., AW-SP, OK-Geo, OK-LU, OK-Soil, OK-Grassland, OK-Geology, OK and IDW) are illustrated in Fig. 1(a–h). The spatial patterns of AW-SP and OK-Geo captured similar major spatial patterns and trends of soil potassium content but had evident smooth surface patterns and could not more accurately describe the local variation in OK-Geo, for which the simulated results ranges were somewhat narrower in the predictions. The OK produced a map similar to those of OK-Geo and AW-SP but with the smoothing effect and weak “bull’s eye” patterns. The predictions of IDW displayed similar major patterns but failed to predict the changes in local variation and displayed strong “bull’s eye” patterns at high and low sample points. The predictions of OK-LU, OK-Soil, OK-Grassland, and OK-Geology combined with environmental variables eliminated the smoothing effect of OK and had significant variation in the different abrupt boundary types. For example, in the soil potassium content interpolation, combining land use information, OK-Landuse gave more details of soil potassium content distribution in different land use types, especially in the abrupt boundary. In the opposite, soil potassium content values of OK and IDW interpolation map did not have the discrete information.

Therefore, the interpolators that considered the environmental variables can more accurately describe the local variation. These results showed that combining appropriate environmental variables (excluding the geology types) as a secondary variable could significantly improve the local variation interpolation performance.

## Discussion

### The performance of ensemble learning for spatial interpolation

Kriging usually outperforms IDW and is generally superior, at least in theory^3^. However, in this study, kriging performed similarly to IDW (e.g., OK) or less well (e.g., OK-Geology). A similar finding was also reported by Collins and Bolstad^12^, who found that optimal IDW was superior to kriging when the data were isotropic and when the primary variable was not correlated with the secondary variable. In this study, even though the correlations between the primary variable and secondary variables were strong, suggesting a strong spatial trend, kringing (e.g., OK-Geology) was not always superior to IDW, at least for soil potassium content.

In contrast to ordinary interpolation methods (e.g., OK) that attempt to generate one learner from the training data, the ensemble method tries to construct a set of base learners and then to combine them. The interpolation accuracy of an ensemble is usually much better than that of a single interpolation model, which makes ensemble methods very attractive. The superior performance of AW-SP in this study could be attributed to the following factors associated with the methods.

The training set of soil potassium content might not provide sufficient information for selection of the single best interpolation model. For example, there might be many interpolation models that perform equally well with the given training set. Thus, combining these interpolation models (e.g., OK-LU, OK-Soil, and OK-Grassland) might be a better choice.

The training set of soil potassium content being interpolated might not contain the true spatial pattern, while the ensembles can provide a good approximation. For example, it is known that for the same piece of rainfed cropland, the soil potassium content for chernozem soil type will be quite different to that of a sandy soil region. Therefore, if land use type is only considered as secondary variables, the use of a single OK-LU method will not lead to a good interpolation, whereas a better approximation could be achieved by combining a set of interpolation methods (e.g., OK-LU, OK-Soil, and OK-Grassland).

The predictions of AW-SP are more reasonable for the extrapolation of soil potassium content in this study and more accurate than OK, IDW, and OK-Geo.

### The effectiveness of secondary variables for reducing predictive error

The distribution of soil properties is controlled by several environmental variables, such as land use, soil type, and slope^33^. The soil property distribution could vary significantly within small spatial scales because of different soil environment types, which can make it difficult to obtain accurate interpolations using AW-SP when such obvious secondary variables are ignored. Therefore, environmental variables should be combined with AW-SP to improve interpolation efficiency.

Different types of land use, soil, geology, slope, and vegetation cover all have an effect on land surfaces. Several studies have indicated that environmental variables are significantly related to the spatial distribution of soil properties^2^,^34^,^35^. Hu *et al*.^35^ and Shi *et al*.^2^ have shown that the spatial distribution pattern of soil properties has strong correlation with different environmental variables^2^,^35^.

A comparison between the accuracy of those methods that use secondary information (i.e., AW-SP, OK-Geo, OK-LU, OK-Soil, and OK-Grassland except OK-Slope and OK-Geology) and that of the methods that do not use secondary information (i.e., OK and IDW) shows that the use of secondary information improves the accuracy of the interpolation. This finding is consistent with previous studies that have shown that stronger correlations result in more accurate predictions when using ordinary cokriging^36^ and ordinary cokriging over OK^37^. It was also reported that a threshold exists because for a correlation >0.4 simple cokriging and ordinary cokriging performed better than simple kriging and OK, for the stronger correlations (r >0.75), the methods those uses the information available on the secondary variable are more superior to OK^38^. However, the OK-Geology and OK-Slope methods are two exceptions, despite the fact that the correlation between soil potassium content and geology type was found to be strong in this study (Table 4). This suggests that the inclusion of secondary information does not always improve the prediction accuracy, which does not support the argument regarding the role of secondary variables in spatial interpolation^2^,^5^,^25^. In this study, this could probably be attributed to the low density of sampling of soil potassium content.

### Limitations

The limitation of AW-SP is that it has a smoothing effect and that the surface variation is smaller than the ensemble object values. If the ensemble results for an object are smaller than the measured value, the AW-SP results will be lower than the measured value. The current research method used ‘tandem’ ensemble interpolation models, incorporating a global interpolation model for the entire study area, although a simple global model cannot explain the spatial instability of soil properties. In future, we will use ‘parallel’ ensemble interpolation models, based on the different regional characteristics of the study area and with consideration of the problems of simulation scale, to select the appropriate interpolation model integration.

## Methods

Section 4.1 describes the AW-SP process. The AW-SP is constructed in two steps. First, a number of base learners are produced (e.g., OK-LU, OK-Soil, and OK-Grassland). Then, the base learners are combined using a popular combination scheme. Section 4.2 shows the interpolation parameter specifications. Section 4.3 shows how to select the secondary variables. Section 4.4 shows how to assess interpolation performance.

### AW-SP

Here we constructed the interpolation model (i.e., OK-LU, OK-Soil, OK-Grassland, and OK-Geology) as the base learner of the AW-SP. As a kind of geostatistical model^31^,^38^, each observation *Z*(*x*_*m,n*_, *y*_*m,n*_) of a specific soil potassium content at location (*x, y*) in the *n-*th type of the *m-*th kind of environmental feature can be expressed as equation (1).


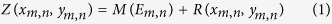


where *M*(*E*_*m,n*_) is the mean value of *Z*(*x*_*m,n*_, *y*_*m,n*_) in the *n-*th type of the *m-*th kind of environmental feature, and *R*(*x*_*m,n*_, *y*_*m,n*_) is the residual computed by subtracting the mean value *M*(*E*_*m,n*_) of the *n-*th type of the relative *m-*th environmental feature from the measured value of soil potassium content. We assume that *M*(*E*_*m,n*_) and *R*(*x*_*m,n*_, *y*_*m,n*_) are mutually independent and that the variation of *R*(*x*_*m,n*_, *y*_*m,n*_) is homogeneous over the entire study area.

**Figure 2:**
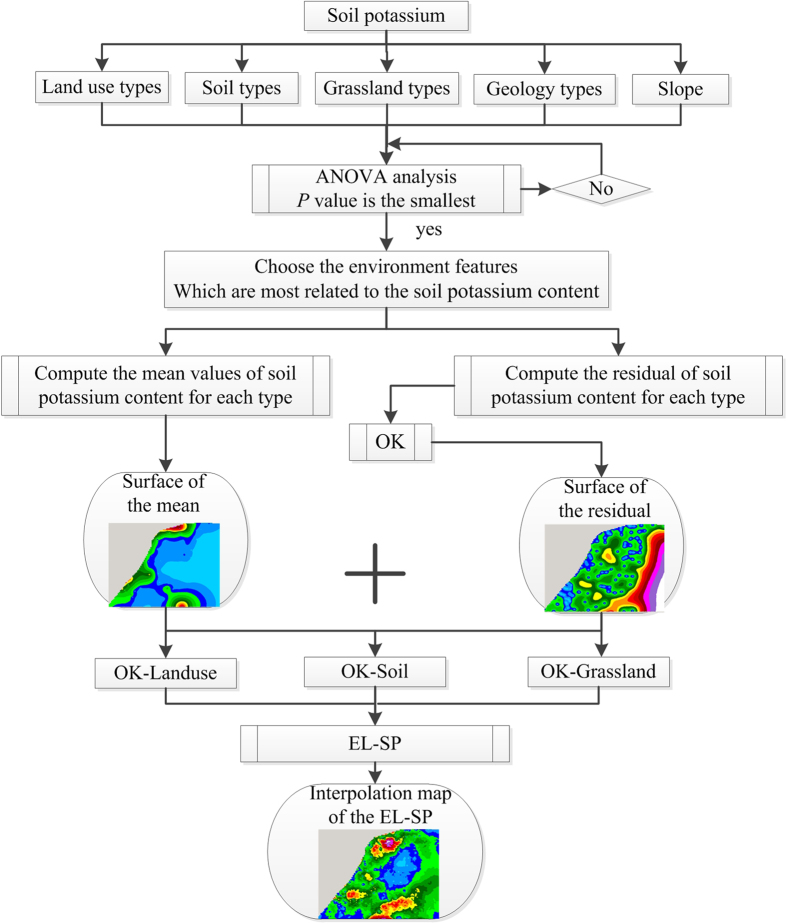
Framework of base learner of AW-SP.

The residuals of the relevant types of environment features are then used to interpolate the surface of the residuals in the entire study area by OK. The interpolated values of residuals are finally summed to the mean values of soil potassium content as one base learner of AW-SP. The construction of base learner framework is shown in Fig. 2 and the steps are described in the below.

- (1) Based on the ANOVA analysis, we analyzed the environmental features that affected the spatial distribution pattern of soil potassium content most significantly and chose the environmental features (*m*) which was most related to soil potassium content.
- (2) Based on the measured values of soil potassium content, we calculated the mean and residuals of the soil potassium content values for each type (*n*) of environmental feature (*m*).
- (3) According to the spatial distribution pattern of the mean values of soil potassium content related to the environmental features (*m*), we converted the environmental features (*m*) to 30 m resolution grids according to the mean values of each type (*n*) using the modules of the Conversion Tools of ArcGIS 10.1, and mapped the overall distribution pattern of soil potassium content *M*(*E*_*m,n*_) for each type (*n*).
- (4) Based on the related residuals, we used OK to simulate the remaining residual surface of soil potassium content *R*(*x*_*m,n*_, *y*_*m,n*_).
- (5) We added the mean surface and residual surface to obtain the *m-*th environmental feature related to the interpolation surface (e.g., OK-LU) as the ensemble learning of the base learners.

Second, the ensemble learning framework algorithm (see Supplementary Methods) was used to integrate all of the interpolation models (e.g., OK-LU, OK-Soil, and OK-Grassland) as the AW-SP simulation result. The steps are described below.

- (1) The ensemble learning algorithm assigned equal weights to all the sampling points of the training soil potassium content data, and the distribution of the weights was denoted at the *t*-th learning round as *D*_*t*_.
- (2) From the training dataset and *D*_*t*_, the ensemble learning framework algorithm chooses a base learner *h*_*t*_ (e.g., OK-LU) by calling the base learning algorithm.
- (3) Then, it used verification points to test *h*_*t*_ (see Supplementary Methods the equation 1) and increased the weights of incorrectly interpolated points. Thus, an updated weight distribution *D*_*t*+1_ was obtained (see Supplementary Methods the equation 3).
- (4) From the training dataset and *D*_*t*+1_, the algorithm used another base learner (e.g., OK-Soil) by calling the base learning algorithm again.
- (5) Such a process was repeated *T* times (*T* depends on the number of base learner) and the final learner derived by weighted averaging of the *T* base learners, where the weights of the learners were determined during the training process (see Supplementary Methods the equation 2).

### Parameter specification

**Figure 3:**
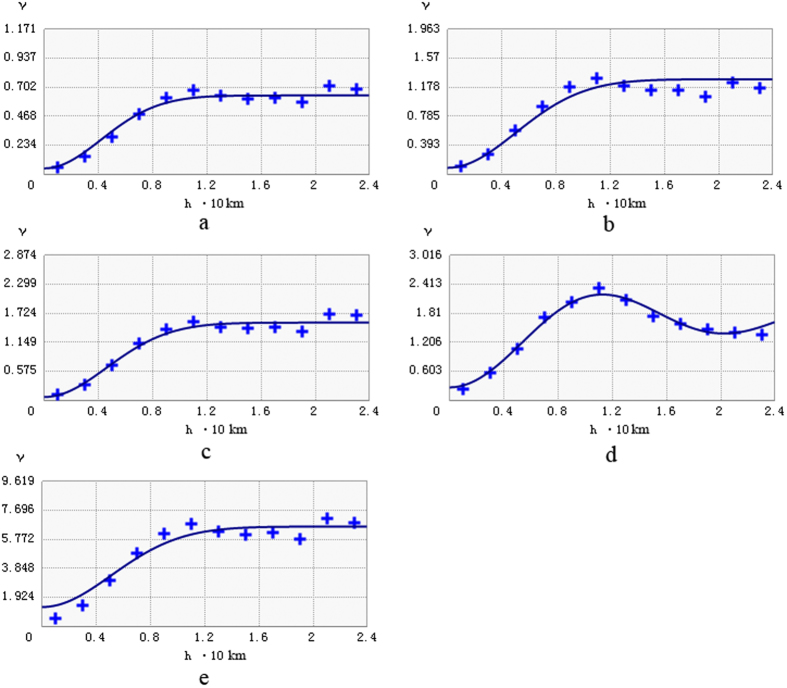
Semi-variograms of original values and residuals for Soil Potassium Content: (**a**) OK-LU, (**b**) OK-Soil, (**c**) OK-Grassland, (**d**) OK-Geology and (**e**) OK.

The parameter specifications were based on the requirements of the interpolation methods and data characteristics. Based on the fitted values of range, nugget, and sill, the variogram model was selected from a series of models including Spherical, Exponential, Gaussian, Hole effect, and Linear models. For OK and its combined methods, the Gaussian and J-Bessel models were selected as they better fitted the data and the residuals of the relevant methods than other variogram models in terms of range, nugget, and sill (Fig. 3 and Table 5). We chose the best kriging sample from 5 to 30 with five-step intervals. IDW was estimated with powers of 1, 2, 3, and 4.

Analyses of the spatial correlation of residuals reflected good performance after removing the local mean within the different secondary variables (Fig. 3). All of the semi-variograms of the residuals tended to show a shorter range and a smaller sill, which indicated that the drift had indeed been removed^27^. All of the N/S (except OK) were <0.3, indicating that the mean sample data has strong spatial correlation^39^, after trend removal, the spatial correlation was stronger (Table 5). This finding suggests that the use of OK and its combined methods is appropriate for this study region.

### Secondary variable selection

Analysis of variance (ANOVA) was performed to analyze the significance of the secondary variables in soil potassium content (Table 4). Take land use types for example: To compare the difference of soil potassium content among land use types, the soil potassium content data were grouped into seven classes based on main land use type. There were 11, 78, 15, 36, three, one, and four samples from rainfed cropland, natural grazing land, tame grassland, other land, scrubland, other grassland, and sandy land, respectively. The variances of each soil potassium content between and within land use types were determined using ANOVA with the software package SPSS 21.0 for Windows. It was established that land use, soil, grassland, and geology types were the four strongest variables correlated with soil potassium content (all significant at the 0.01 level). Based on intuition and other references^22^,^40^, it was considered likely that slope would have some influence on the transfer of soil potassium content from the high-slope regions. Therefore, slope was also considered as an important secondary variable in this study. However, slope was eventually excluded because of its lack of correlation with soil potassium content (The slope varied only slightly across the study area, most slopes were between 0° and 8°).With the exception of the geology type, they were used as secondary variables in the AW-SP and OK-Geo methods. Geology was dropped because its performance when combined with OK was worse than OK alone, despite the strong correlation found in this study between soil potassium content and geology type. The types of land use, soil, grassland, and geology were used in the OK-LU, OK-Soil, OK-Grassland, and OK-Geology methods, respectively. The OK and IDW methods do not need secondary variables.

### Assessment of the performance

Independent verification was used for the validation of the interpolators in this study. The procedure involves randomly splitting the data into the interpolation and validation subsets, estimating the value using interpolation subset and comparing the interpolated value at every validation point with its measured value. A total of 120 training points were randomly created as interpolation data sets, and the remaining 28 samples were used as validation data sets.

The performance of these interpolation methods was assessed by identifying the error in the predictions. We used the three most common indices, i.e., the mean absolute error (MAE), the root mean square error (RMSE) and the mean error (ME) as measures of the interpolation quality. The formulations of the MAE, RMSE and ME are as below equation (2), (3) and (4).


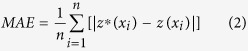



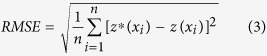



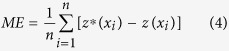


where z*(*xi*) is the measured value, *z*(*xi*) is the predicted value, and n is the number of validation points. The values of the two indices should be close to zero if the method is completely accurate. In comparison, RMSE is sensitive to the size of outliers and it is used as an indicator of the magnitude of extreme errors. Lower values of RMSE indicate greater central tendency and generally smaller extreme errors^41^.

## Data and Study Area

### Study area

**Figure 4:**
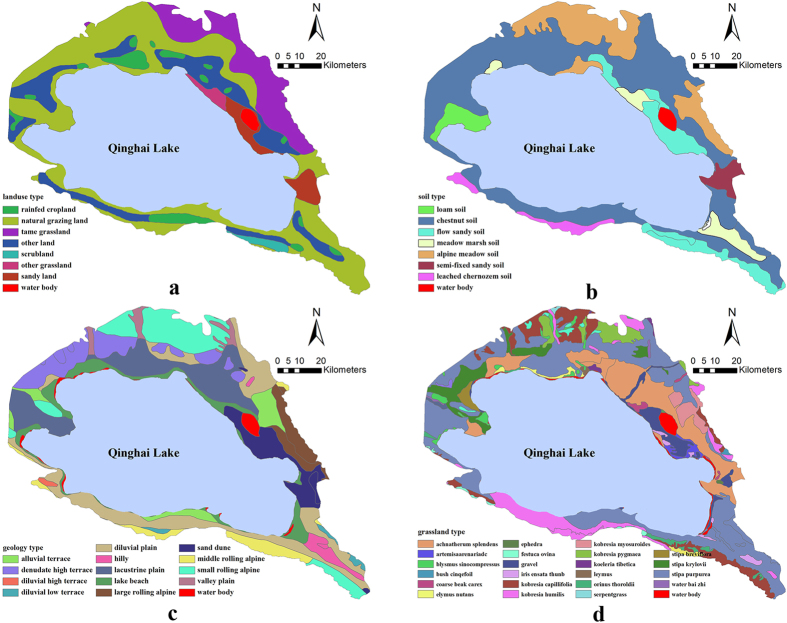
Environmental variables of the study area and the distribution of soil samples. (**a**) land use types, (**b**) soil types, (**c**) geology types and (**d**) grassland types. All the maps were generated in ArcGIS10.1, URL: http://www.esrichina-bj.cn/softwareproduct/ArcGIS/.

The study area (36°27′51″–37°30′43″N, 99°55′29″–101°05′03″E) is located in the southeast region of the Qinghai Lake Basin. The region covers an area of 7425.61 km^2^ of which 4473.96 km^2^ is water and the land elevation ranges from 3043 to 4516 m. According to the 1:1,000,000 soil maps of the National Soil Census Office, there are eight groups of soil type (Fig. 4b), and according to the 1:500,000 geological map of Qinghai Province from the Qinghai Provincial Bureau of Geology and Mineral Resources, the principal geological type include alluvial terraces, rolling alpine, and valley plain, etc. (Fig. 4c). Land use type are rainfed cropland, natural grazing land, tame grassland, other land, scrubland, other grassland, sandy land and water body, etc. (Fig. 4a), and the grassland can be classified into twenty-three groups (Fig. 4d). Slope type is not considered in this study because of their poor correlation with the soil potassium content (Table 4).

### Datasets

We collected a total of 148 topsoil samples (0–30 cm) from the study area in September 2013. We also recorded the soil sample locations, land use type, soil type, grassland type, geology type, and elevation. We analyzed the landscape in the study area using the spatial stratified sampling method^42^. Each position was sampled three times and the average was recorded as the sample values. Each sample was air-dried and passed through a 2-mm sieve to determine the soil potassium content.

Many environmental variables can be used as secondary variables to improve the performance of spatial interpolation methods as discussed by Li and Heap^3^ and Shi *et al*.^2^. Following a preliminary analysis, the land use, soil, grassland, and geology types were considered important secondary information in this study. The land use, soil, grassland, and geology types were previously used to improve the performance of the spatial interpolators of soil properties^25^. Therefore, the inclusion of such environmental variables was expected to improve the predictions. ANOVA analysis (Table 4) revealed that variances of the tested soil potassium content among different secondary variables (except for slope) might play an important role in their spatial prediction in the study area. All datasets of environmental variables were generated in ArcGIS10.1 and resampled to 30-m resolution wherever necessary. However, the small sample size and uneven spatial distribution of the soil samples (Fig. 4) indicate that sub-setting by feature types, leading to some features without soil samples, cannot provide an adequate soil sample size for modeling; as such, we used the secondary variables to improve the interpolation accuracy.

## Additional Information

**How to cite this article**: Liu, W. *et al*. An Adaptive Weighting Algorithm for Interpolating the Soil Potassium Content. *Sci. Rep.* **6**, 23889; doi: 10.1038/srep23889 (2016).

## Supplementary Material

Supplementary Information

## Figures and Tables

**Table 1:** Methods compared for predicting soil potassium content in this study.

Methods	Abbreviation	Comments
Inverse distance weighting	IDW	With distance power 2 to 4
Ordinary kriging	OK	
OK combined with geographic information	OK-Geo^7^	OK combined with land use types, soil type and grass land type
OK combined with land use	OK-LU	Trend surface using the mean values of each land use classification contains the soil potassium content, OK are adopted to simulate the remaining residuals.
OK combined with soil	OK-Soil	Trend surface using the mean values of each soil classification contains the soil potassium content, OK are adopted to simulate the remaining residuals.
OK combined with grassland	OK-Grassland	Trend surface using the mean values of each grass land classification contains the soil potassium content, OK are adopted to simulate the remaining residuals.
OK combined with geology	OK-Geology	Trend surface using the mean values of each geology classification contains the soil potassium content, OK are adopted to simulate the remaining residuals.
Ensemble learning combined with geographic information	AW-SP	Ensemble learning combined environmental variables (land use type, soil type and grass land type) for soil properties interpolation

(OK-Geo interpolation by Ordinary Cokriging (OCK) model; OK-LU, OK-Soil, OK-Grassland and OK-Geology using equation (1) to predict; AW-SP using OK-LU, OK-Soil and OK-Grassland of interpolation results as the base learner).

**Table 2:** Comparisons of the accuracy among IDW, OK, OK-LU, OK-Soil, OK-Grassland, OK-Geology, OK-Geo and AW-SP.

Methods	MAE	RMSE	ME
IDW	0.1487	0.1872	−0.0030
OK	0.1485	0.1838	0.0026
OK-LU	0.1376	0.1741	0.0017
OK-Soil	0.1381	0.1754	0.0015
OK-Grassland	0.1387	0.1797	0.0012
OK-Geology	0.1521	0.2022	0.0026
OK-Geo	0.1284	0.1732	0.0011
AW-SP	0.1003	0.1374	0.0000

**Table 3:** Effects of slope and geology exclusion on the prediction error of AW-SP and OK-Geo.

Method	Slope	Geology	MAE	p-value	RMSE	p-value	ME	p-value
AW-SP	Yes	No	0.1417	0.0032	0.1781	0.0018	0.0008	0.0043
AW-SP	No	Yes	0.1348	0.0354	0.1707	0.0223	0.0005	0.0438
OK-Geo	Yes	No	0.1614	0.0159	0.2076	0.0376	0.0017	0.0283
OK-Geo	No	Yes	0.1367	0.0251	0.1873	0.0247	0.0012	0.0326

Paired t-test was used to examine if the predictive errors (i.e., MAE, RMSE and ME) of methods with slope or geology are greater than those without slope or geology based on the results of independent verification.

**Table 4:** ANOVA for testing the effects of secondary variables on variances of soil potassium.

Methods	Secondaryvariables	Source ofvariance	Sum ofsquares	df	Meansquare	F	Sig.
AW-SP	Land use type	Between	1.471	5	0.294	7.785	<0.01
OK-Geo		Within	5.177	143	0.038		
OK-LU		Total	6.648	148			
AW-SP	Soil type	Between	1.549	6	0.258	6.886	<0.01
OK-Geo		Within	5.099	142	0.037		
OK-Soil		Total	6.648	148			
AW-SP	Grassland type	Between	1.237	13	0.273	7.800	<0.01
OK-Geo		Within	5.411	135	0.035		
OK-Grassland		Total	6.648	148			
AW-SP	Geology type	Between	2.813	11	0.256	8.738	<0.01
OK-Geo		Within	3.835	137	0.029		
OK-Geology		Total	6.649	148			
	Slope type	Between	0.071	4	0.036	0.878	0.09
Restricted		Within	6.577	144	0.041		
		Total	6.648	148			

**Table 5:** Semi-variagram models.

Parameter	Residue ofOK_LU	Residue ofOK_Soil	Residue ofOK_Grassland	Residue ofOK_Geology	OK
Model	Gaussian	Gaussian	Gaussian	J-Bessel	Gaussian
Range/10 km	1.0300	1.2105	1.1130	1.6213	1.3623
Nugget(*N*)	0.0801	0.0913	0.1237	0.2931	1.8524
Sill(*S*)	0.5236	1.1832	1.6123	1.6621	5.8121
*N/S*	0.1527	0.0772	0.0767	0.1763	0.3187
