# Supplementary material for: An Adaptive Weighting Algorithm for Interpolating the Soil Potassium Content
Source: Sci Rep. 2016 Apr 7;6:23889. doi: 10.1038/srep23889 (PMC4823722; doi:10.1038/srep23889)
Supplement: Supplementary Information [file srep23889-s1.doc]

**An Adaptive Weighting Algorithm for Interpolating the Soil Potassium Content**

**Wei Liu,** **Peijun Du, Zhuowen Zhao, Lianpeng Zhang**

| **Input:**  // *x* is measured value, *X* is measured values set; y is predicted value, *Y* is predicted values set.  Data set D= {(*x_1_, y_1_*), (*x_2_, y_2_*)... (*x_m_, y_m_*)} where, *x* ∈*X*, *y*∈*Y*;  // *h* is the interpolation model set (e.g., OK-LU, OK-Soil and OK-Grassland).  Base learner *h;*  Number of learning rounds *t*.  **Process:**  //Initialize the weight distribution of each sampling point.  *D1(i)*=1/*m*  // *t* depends on the number of base learner, if there are *l* base learners, the *T*= *l.*  *for t =1，…， T*  //Measure the interpolation error of *h_t_*. where I [*] is the indication function which outputs 1 if the inner expression is true and 0 otherwise.$h_{t}(\frac{y_{i}}{x_{i}}<0.95)$ is under the *D_t_* distribution using the *h_t_* model, whether a certain sample point interpolation precision less than 0.95, i.e., 0.95 is a threshold value, which can be set according to demand. $\varepsilon_{t}=pr_{i\sim D_{i}}I[h_{t}(\frac{y_{i}}{x_{i}}<0.95)](1)$   // If an interpolation model prediction error rate is more than 50%, drop the model.  *if ε_t_*≥*1/2*, then stop;  // Determine the weight of *h_t_*.  *Set* $a_{t}=\frac{1}{2}\ln(\frac{1-\varepsilon_{t}}{\varepsilon_{t}})(2)$ // Update the distribution, where *Zt* is a normalization factor which enables *Dt+1* to be a distribution. $D_{t+1}(i)=\frac{D_{t}(i)}{z_{t}}\times\left\{\begin{matrix} \exp(-a_{t})ifh_{t}(\frac{y_{i}}{x_{i}}\geq0.95) \\ \exp(a_{t})ifh_{t}(\frac{y_{i}}{x_{i}}<0.95) \end{matrix}\right.(3)$   End  **Output:**  The final interpolation function: $H(\mathrm{x})=\sum_{t=1}^{T} w_{t}h_{t}(x)$ Where $w_{t}$is a normalization factor: $w_{t}=\frac{a_{t}}{\sum_{t=1}^{T} a_{t}}$ |
| --- |

Supplementary Method. Pseudo-code of AW-SP method
